# *Papiliocoelotes* gen. n., a new genus of Coelotinae (Araneae, Agelenidae) spiders from the Wuling Mountains, China

**DOI:** 10.3897/zookeys.585.8007

**Published:** 2016-04-27

**Authors:** Zhe Zhao, Shuqiang Li

**Affiliations:** 1 Institute of Zoology, Chinese Academy of Sciences, Beijing 100101, China; 2 University of the Chinese Academy of Sciences, Beijing 100049, China

**Keywords:** Taxonomy, coelotine, description, diagnosis, Asia

## Abstract

One new genus of the spider subfamily Coelotinae, *Papiliocoelotes* **gen. n.**, with five new species is described for both sexes: *Papiliocoelotes guanyinensis* **sp. n.**, *Papiliocoelotes guitangensis* **sp. n.**, *Papiliocoelotes jiepingensis* **sp. n.**, *Papiliocoelotes meiyuensis* **sp. n.**, *Papiliocoelotes yezhouensis* **sp. n.** All new species were collected from caves in the Wuling Mountains of Hubei and Hunan Provinces, China. DNA barcodes were obtained for future use.

## Introduction

Coelotine spiders are only distributed in the temperate and tropical areas of the Northern Hemisphere. So far, a total of 657 valid species belonging to 24 genera (Wang 2012, Chen et al. 2015b, Chen et al. 2016) are known, and 18 genera are distributed in China. Wang (2002) erected 10 new genera, and more than half of them are primarily distributed in southern China. This distribution shows that the potential generic-level diversity of coelotine spiders is yet to be discovered (although three of those genera are considered synonyms and no longer used) (World Spider Catalog 2016). In the last three years, a new genus from southern China (Chen et al. 2015b) and many new coelotine species were described successively from China (Chen et al. 2015a, Jiang and Chen 2015) and adjacent regions: Caucasus (Kovblyuk et al. 2013), Japan (Okumura 2013) and Korea (Kim and Ye 2013, Kim and Ye 2014, Seo 2014, Ye and Kim 2014), suggesting that there are still many poorly known species and genera in those areas.

In this study, a new genus of coelotine spider, *Papiliocoelotes* gen. n. and five new species from Hubei and Hunan Provinces in southern China are reported.

## Material and methods

Specimens were examined with a LEICA M205C stereomicroscope. Images were captured with an Olympus C7070 wide zoom digital camera (7.1 megapixels) mounted on an Olympus SZX12 dissecting microscope. Epigynes and male palps were examined after dissection from the spiders’ bodies.

All measurements were obtained using a LEICA M205C stereomicroscope and are given in millimeters. Leg measurements are shown as: total length (femur, patella + tibia, metatarsus, tarsus). Only structures (palp and legs) on the left side of the body were described and measured. The abbreviations and terminology used in the text follows Wang (2002). The pattern was not described for each species because it is shown in the figures and is nearly the same in all species. Abbreviations used in this paper and in the figure legends: ALE = anterior lateral eye; AME = anterior median eye; AME-ALE = distance between AME and ALE; AME-AME = distance between AME and AME; ALE-PLE = distance between ALE and PLE; BH = basal hematodocha; C = conductor; CD = copulatory duct; CO = copulatory opening; CF = cymbial furrow; E = embolus; EB = embolic base; FD = fertilization duct; H = epigynal hood; PA = patellar apophysis; PLE = posterior lateral eye; PME = posterior median eye; PME-PLE = distance between PME and PLE; PME-PME = distance between PME and PME; RTA = retroventral tibial apophysis; S = spermatheca; ST = subtegulum; T = tegulum; TA = tegular apophysis; PC = patellar condyle.

DNA barcodes were obtained for future use. A partial fragment of the mitochondrial gene cytochrome oxidase subunit I (COI) was amplified and sequenced for *Papiliocoelotes guanyinensis* sp. n., *Papiliocoelotes guitangensis* sp. n., *Papiliocoelotes jiepingensis* sp. n., *Papiliocoelotes meiyuensis* sp. n. and *Papiliocoelotes yezhouensis* sp. n. using Primers LCO1490-oono (5’-CWACAAAYCATARRGATATTGG-3’) (Folmer et al. 1994; Miller et al. 2010) and HCO2198-zz (5’-TAAACTTCCAGGTGACCAAAAAATCA-3’) (Folmer et al. 1994; Chen et al. 2015a). For additional information on extraction, amplification and sequencing procedures, see Zhao et al. (2013). All sequences were analyzed using BLAST and are deposited in GenBank. The accession numbers are provided in Table 1.

**Table 1.** Voucher specimen information.

Species	GenBank accession number	Sequence length	Collection localities
*Papiliocoelotes guanyinensis* sp. n.	KU991801	630 bp	Hefeng County, Enshi Prefecture, Hubei, China
*Papiliocoelotes guitangensis* sp. n.	KU991804	630 bp	Longshan County, Hunan, China
*Papiliocoelotes jiepingensis* sp. n.	KU991803	630 bp	Xianfeng County, Enshi Prefecture, Hubei, China
*Papiliocoelotes meiyuensis* sp. n.	KU991802	630 bp	Hefeng County, Enshi Prefecture, Hubei, China
*Papiliocoelotes yezhouensis* sp. n.	KU991800	627 bp	Jianshi County, Enshi Prefecture, Hubei, China

All species were collected from caves in the Wuling Mountains. All specimens (including molecular vouchers) are deposited in the Institute of Zoology, Chinese Academy of Sciences in Beijing (IZCAS).

## Systematics

### Family Agelenidae C.L. Koch, 1837 Subfamily Coelotinae F.O.P.-Cambridge, 1893

#### Genus Papiliocoelotes gen. n.

Taxon classificationAnimaliaAraneaeAgelenidae

http://zoobank.org/223E0874-B0AF-413C-879D-9BB08DC8CF4E

##### Type species.

*Papiliocoelotes yezhouensis* sp. n.

##### Etymology.

The generic name is derived from the Latin word “Papilio”, meaning “butterfly, moth”, referring to the shape of the endogyne, and “Coelotes” referring to the similarity with the nominal genus of the subfamily. The gender is masculine.

##### Diagnosis.

**Figure 1.**
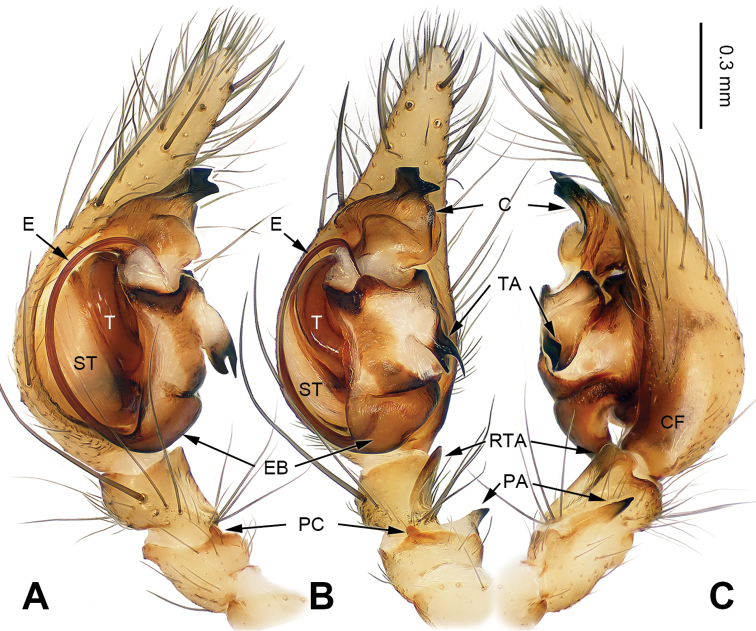
Left male palp of *Papiliocoelotes yezhouensis* sp. n., holotype. **A** Prolateral view **B** Ventral view **C** Retrolateral view. Scale bar: equal for **A**, **B**, **C**.

**Figure 2.**
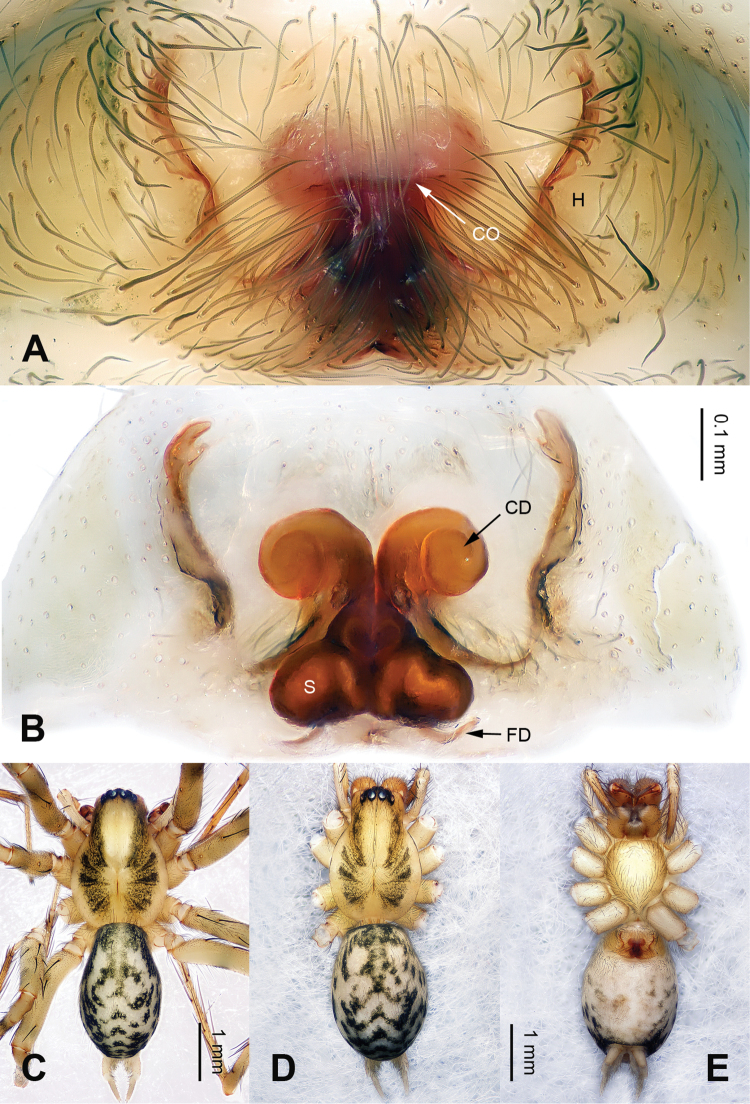
Epigyne and habitus of *Papiliocoelotes yezhouensis* sp. n. **A** Epigyne, ventral **B** Vulva, dorsal **C** Male habitus, dorsal **D** Female habitus, dorsal **E** Female habitus, ventral. Scale bars: equal for **A** and **B**, equal for **D** and **E**.

Males can be easily distinguished from other coelotines, except *Platocoelotes* Wang, 2002, by the absence of a median apophysis and the presence of an elongated tegular apophysis. They can be distinguished from *Platocoelotes* by the broad conductor without the embolus inside and the relatively short embolus that terminates at the base of conductor (Fig. 1A–C; Wang 2002: figs 338, 339; Xu and Li 2008: figs 11–16; Chen et al. 2015a: fig. 1A–C). Females can be easily distinguished from other coelotines, except *Platocoelotes* and *Spiricoelotes* Wang, 2002, by having no epigynal teeth and the presence of epigynal hoods. They can be distinguished from *Platocoelotes* by the shape of the copulatory ducts, which are weakly sclerotized and spiraled, whereas the copulatory ducts are usually broad in *Platocoelotes* (Fig. 2A–B; Chen et al. 2015a). They can distinguished from *Spiricoelotes* by the positions of the epigynal hoods that are located mediolaterally or posterolaterally on the epigynal plate, whereas the epigynal hoods are usually located anterolaterally in *Spiricoelotes*, and by the sclerotized and spiral copulatory ducts (Fig. 2A–B; Wang 2002: figs 360, 361; Chen et al. 2016: fig. 2A–B).

##### Description.

Small to medium-sized, with a total length of 4–7 mm; body color is shallow, with black stripes; carapace nearly pear-shaped, with radial pattern; sternum yellowish; abdomen nearly oval-shaped, with herringbone pattern in dorsal view; chelicerae usually with 1 to 3 promarginal and 2 retromarginal teeth in both sexes; leg formula (4 > 1 > 2 ≥ 3). Male palp with 1 patellar apophysis and 1 patellar condyle; retroventral tibial apophysis extending beyond the distal margin of tibia; conductor broad; tegulum with tegular apophysis; embolus filiform, relatively short and terminates at the base of conductor. Epigynal teeth absent; atrium usually small or indistinct; epigynal hoods located mediolaterally or posterolaterally; copulatory openings usually located centrally or posterior centrally on the epigyne plate; the shape of spermathecae and copulatory ducts butterfly-like; spermathecae located in posterior of epigyne; spermathecal head indistinct; copulatory ducts sclerotized and spiral.

##### Comments.

In addition to morphological study, we reconstructed the phylogeny of coelotine spiders based on molecular data from 18 genera and 286 coelotine species (the phylogenetic analysis results will be published in a subsequent paper). The molecular phylogenetic analyses support *Papiliocoelotes* as monophyletic and closely related to *Platocoelotes* and *Spiricoelotes*.

##### Distribution.

China (Hubei, Hunan) (Fig. 11).

#### Papiliocoelotes yezhouensis sp. n.

Taxon classificationAnimaliaAraneaeAgelenidae

http://zoobank.org/5466FCB3-9F26-4FE7-A40E-5B77283191E6

Figs 1 , 2 , 11

##### Type material.

**Holotype** ♂: China: Hubei: Enshi Prefecture: Jianshi County: Yezhou Town: near gas station, a unnamed cave (near a sandpit), N30.63685°, E109.72212°, 588 m, 21.I.2014, Y. Li and J. Liu leg. **Paratypes**: 4♀2♂, same data as holotype.

##### Etymology.

The specific name is derived from the type locality; adjective.

##### Diagnosis.

The male can be distinguished from *Papiliocoelotes meiyuensis* sp. n. by the short and wide tegular apophysis with a bifurcated tip, the broad conductor with a slightly bifurcated distal process and a long patellar condyle (Fig. 1A–C). The female can be distinguished from *Papiliocoelotes meiyuensis* sp. n. by the thick copulatory ducts that roll into a circle (Fig. 2A–B).

##### Description.

**Male (holotype)**: Total length 4.85. Carapace 2.50 long, 1.75 wide. Abdomen 2.40 long, 1.60 wide. Eye sizes and interdistances: AME 0.07, ALE 0.11, PME 0.13, PLE 0.13; AME-AME 0.05, AME-ALE 0.03, ALE-PLE 0.05, PME-PME 0.05, PME-PLE 0.07. Leg measurements: I: 9.45 (2.50, 3.15, 2.30, 1.50); II: 8.20 (2.25, 2.65, 1.95, 1.35); III: 7.85 (2.10, 2.45, 2.10, 1.20); IV: 10.60 (2.75, 3.25, 3.10, 1.50). Chelicerae with 3 promarginal teeth. Palp: patellar apophysis long, scarcely curved, with pointed tip, extending anteriorly; patellar condyle long; retroventral tibial apophysis almost rectangular apically; cymbial furrow broad and about 2/5 length of cymbium; conductor broad, blunt apically; conductor with slightly bifurcated distal process; tegular apophysis short with bifurcated tip, shorter than the length of the cymbial furrow (Fig. 1A–C).

**Female (one of paratypes)**: Total length 5.05. Carapace 2.45 long, 1.60 wide. Abdomen 2.45 long, 1.70 wide. Eye sizes and interdistances: AME 0.07, ALE 0.11, PME 0.12, PLE 0.12; AME-AME 0.05, AME-ALE 0.02, ALE-PLE 0.03, PME-PME 0.05, PME-PLE 0.07. Leg measurements: I: 8.15 (2.25, 2.80, 1.85, 1.25); II: 7.00 (2.00, 2.25, 1.60, 1.15); III: 6.65 (1.85, 2.00, 1.75, 0.95); IV: 9.00 (2.40, 2.85, 2.50, 1.25). Chelicerae as in male. Epigyne: copulatory openings located centrally; epigynal hoods located mediolaterally, sulci small and shallow; copulatory ducts roll into a circle; width of spermathecae subequal to width of copulatory ducts (Fig. 2A–B).

##### Distribution.

Known only from the type locality (Fig. 11).

#### Papiliocoelotes guanyinensis sp. n.

Taxon classificationAnimaliaAraneaeAgelenidae

http://zoobank.org/50C51224-FA28-48AA-A419-646C7075C840

Figs 3 , 4 , 11

##### Type material.

**Holotype** ♂: China: Hubei: Enshi Prefecture: Hefeng County: Guanyinping, Guanyin Cave, N29.93238°, E110.05344°, 758 m, 11.I.2014, Y. Li and J. Liu leg. **Paratypes**: 3♀2♂, same data as holotype.

##### Etymology.

The specific name is derived from the type locality; adjective.

##### Diagnosis.

**Figure 3.**
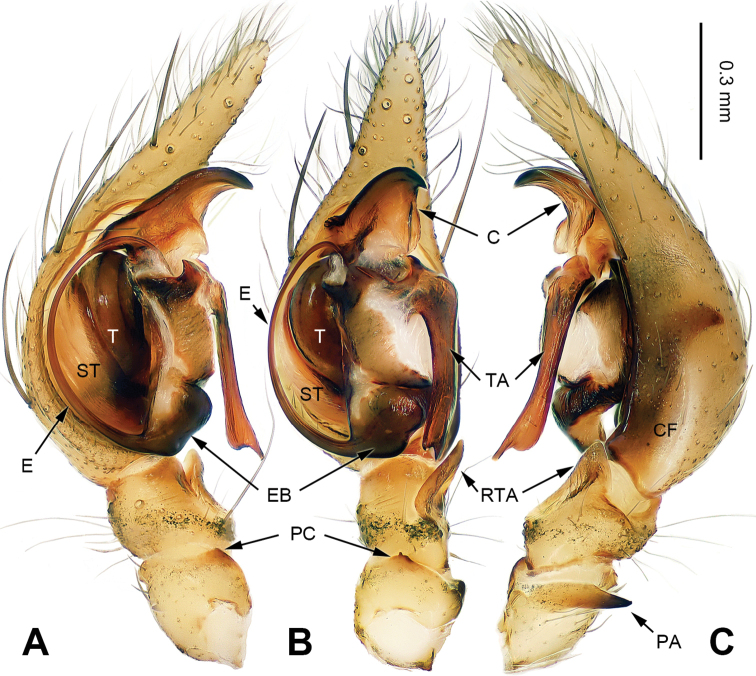
Left male palp of *Papiliocoelotes guanyinensis* sp. n., holotype. **A** Prolateral view **B** Ventral view **C** Retrolateral view. Scale bar: equal for **A, B, C**.

**Figure 4.**
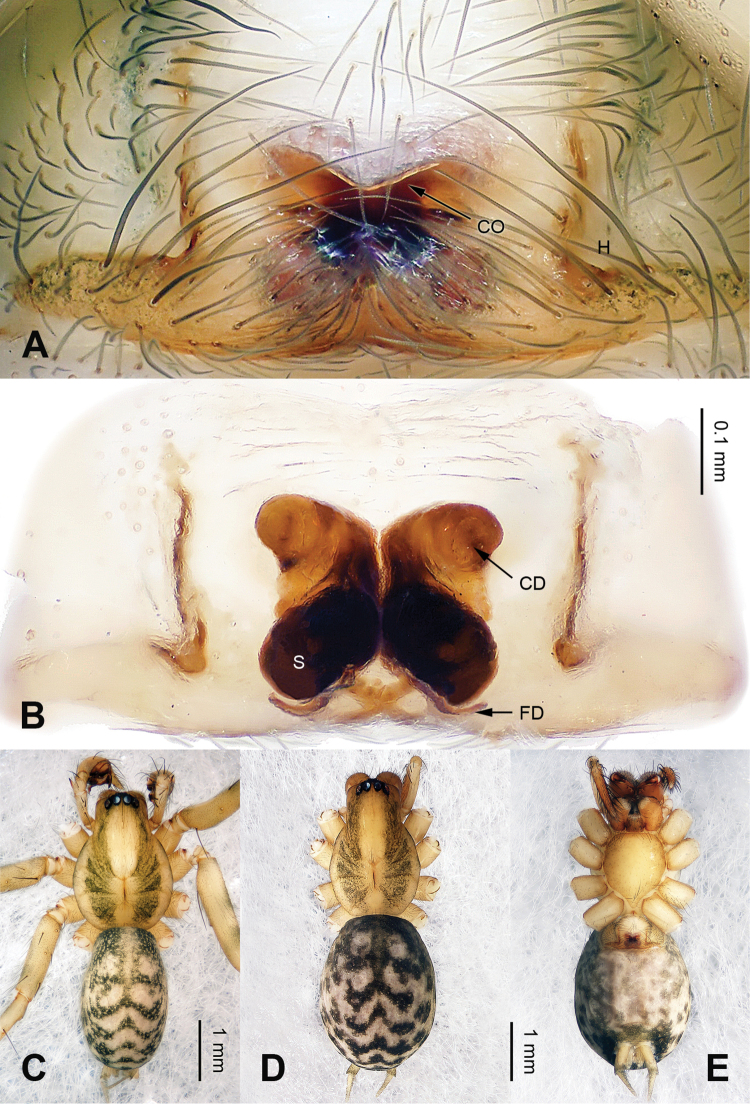
Epigyne and habitus of *Papiliocoelotes guanyinensis* sp. n. **A** Epigyne, ventral **B** Vulva, dorsal **C** Male habitus, dorsal **D** Female habitus, dorsal **E** Female habitus, ventral. Scale bars: equal for **A** and **B**, equal for **D** and **E**.

The male can be distinguished from *Papiliocoelotes yezhouensis* sp. n. by the large tegular apophysis that is longer than the length of the cymbial furrow, the lack of a patellar condyle, the fin-shaped conductor and the dorsally extending patellar apophysis (Fig. 3A–C). The female can be distinguished from *Papiliocoelotes yezhouensis* sp. n. by the distinct copulatory openings and the epigynal hoods which are located posterolaterally (Fig. 4A–B).

##### Description.

**Male (holotype)**: Total length 4.55. Carapace 2.25 long, 1.65 wide. Abdomen 2.35 long, 1.55 wide. Eye sizes and interdistances: AME 0.06, ALE 0.12, PME 0.13, PLE 0.13; AME-AME 0.05, AME-ALE 0.01, ALE-PLE 0.01, PME-PME 0.04, PME-PLE 0.03. Leg measurements: I: 8.60 (2.25, 2.85, 2.00, 1.50); II: 7.40 (2.00, 2.40, 1.85, 1.15); III: 7.10 (1.95, 2.25, 1.75, 1.15); IV: 10.00 (2.60, 3.05, 2.90, 1.45). Chelicerae with 1 promarginal tooth. Palp: patellar apophysis long, scarcely curved, with pointed tip, extending dorsally; patellar condyle absent, only dark distally; retroventral tibial apophysis sharply pointed; cymbial furrow short and indistinct, about 1/3 length of cymbium; conductor fin-shaped; tegular apophysis elongate, slightly blunt at subdistal part and longer than the length of the cymbial furrow (Fig. 3A–C).

**Female (one of paratypes)**: Total length 4.75. Carapace 2.15 long, 1.50 wide. Abdomen 2.65 long, 1.95 wide. Eye sizes and interdistances: AME 0.07, ALE 0.10, PME 0.12, PLE 0.13; AME-AME 0.04, AME-ALE 0.02, ALE-PLE 0.03, PME-PME 0.05, PME-PLE 0.04. Leg measurements: I: 6.90 (2.00, 2.25, 1.45, 1.20); II: 6.26 (1.76, 2.00, 1.50, 1.00); III: 6.15 (1.75, 1.95, 1.45, 1.00); IV: 8.75 (2.00, 2.35, 2.25, 1.15). Chelicerae with 3 promarginal teeth. Epigyne: copulatory openings located centrally; epigynal hoods located posterolaterally, sulci round and deep; copulatory ducts thick and curled; the width of spermathecae subequal to the width of the copulatory ducts (Fig. 4A–B).

##### Distribution.

Known only from the type locality (Fig. 11).

#### Papiliocoelotes guitangensis sp. n.

Taxon classificationAnimaliaAraneaeAgelenidae

http://zoobank.org/951855FC-F477-4DA1-8AAB-043D7726349E

Figs 5 , 6 , 11

##### Type material.

**Holotype** ♂: China: Hunan: Longshan County: Guitangba Town: Wulongshan Park, Feihu Cave, N29.21000°, E109.30569°, 436 m, 13.I.2014, Y. Li and J. Liu leg. **Paratypes**: 2♀2♂, same data as holotype.

##### Etymology.

The specific name is derived from the type locality; adjective.

##### Diagnosis.

**Figure 5.**
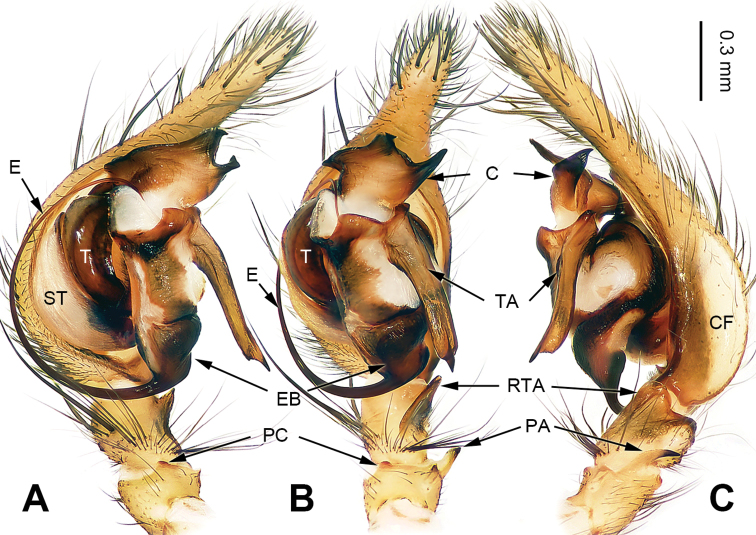
Left male palp of *Papiliocoelotes guitangensis* sp. n., holotype. **A** Prolateral view **B** Ventral view **C** Retrolateral view. Scale bar: equal for **A**, **B**, **C**.

**Figure 6.**
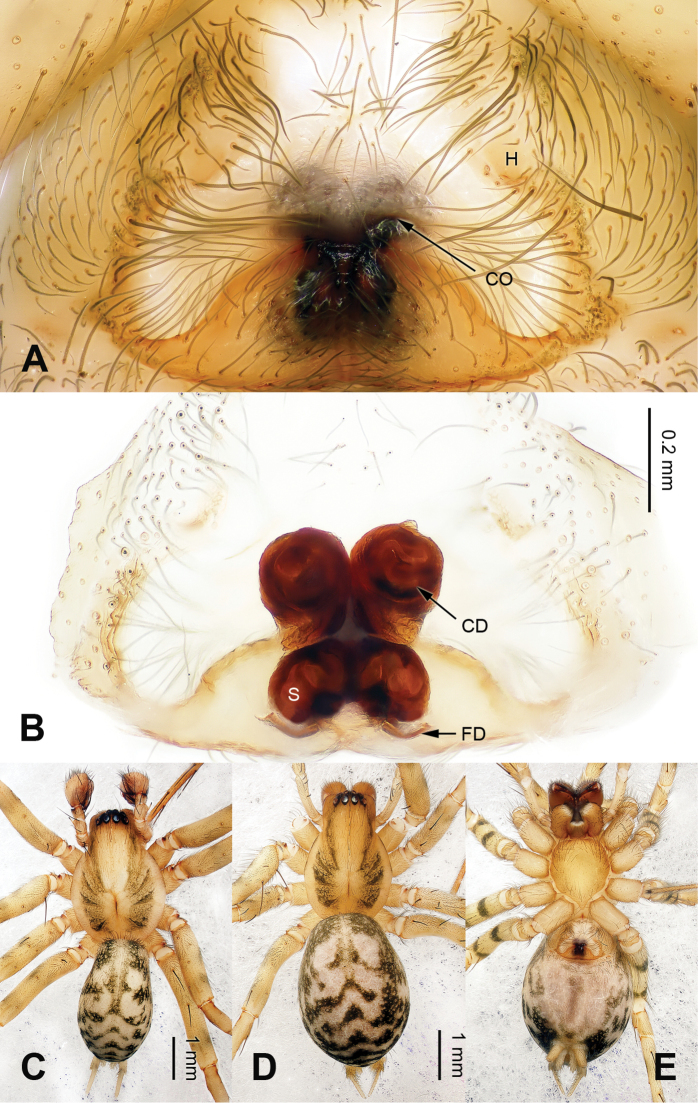
Epigyne and habitus of *Papiliocoelotes guitangensis* sp. n. **A** Epigyne, ventral **B** Vulva, dorsal **C** Male habitus, dorsal **D** Female habitus, dorsal **E** Female habitus, ventral. Scale bars: equal for **A** and **B**, equal for **D** and **E**.

The male can be distinguished from *Papiliocoelotes yezhouensis* sp. n. by the dorsally curved patellar apophysis, the apically rounded retroventral tibial apophysis, the large tegular apophysis with pointed tip and the conductor with 2 pointed distal processes (Fig. 5A–C). The female can be distinguished from *Papiliocoelotes yezhouensis* sp. n. by the small and shallow epigynal hoods, and the width of the copulatory ducts is slightly wider than the spermathecae (Fig. 6A–B).

##### Description.

**Male (holotype)**: Total length 6.05. Carapace 3.25 long, 2.20 wide. Abdomen 2.85 long, 1.75 wide. Eye sizes and interdistances: AME 0.11, ALE 0.14, PME 0.14, PLE 0.15; AME-AME 0.05, AME-ALE 0.02, ALE-PLE 0.03, PME-PME 0.06, PME-PLE 0.08. Leg measurements: I: 11.55 (2.85, 4.00, 2.70, 2.00); II: 10.30 (2.80, 3.25, 2.50, 1.75); III: 8.95 (2.60, 3.00, 2.25, 1.10); IV: 13.05 (3.25, 4.00, 3.80, 2.00). Chelicerae with 1 promarginal tooth. Palp: patellar apophysis long, curved dorsally, with pointed tip; patellar condyle short; retroventral tibial apophysis rounded apically; cymbial furrow broad, about 2/5 length of the cymbium; conductor wide, with 2 pointed distal processes; tegular apophysis elongate with pointed tip, subequal to the length of the cymbial furrow (Fig. 5A–C).

**Female (one of paratypes)**: Total length 5.85. Carapace 2.75 long, 1.80 wide. Abdomen 3.15 long, 2.25 wide. Eye sizes and interdistances: AME 0.11, ALE 0.13, PME 0.13, PLE 0.14; AME-AME 0.04, AME-ALE 0.03, ALE-PLE 0.03, PME-PME 0.05, PME-PLE 0.08. Leg measurements: I: 9.15 (2.50, 3.05, 2.15, 1.45); II: 7.95 (2.20, 2.50, 1.95, 1.30); III: 7.75 (2.05, 2.45, 1.95, 1.30); IV: 10.30 (2.70, 3.25, 2.90, 1.45). Chelicerae with 3 promarginal teeth. Epigyne: copulatory openings located centrally; epigynal hoods small, located mediolaterally, sulci small and shallow; copulatory ducts roll into a ball; the width of copulatory ducts slightly wider than the spermathecae (Fig. 6A–B).

##### Distribution.

Known only from the type locality (Fig. 11).

#### Papiliocoelotes jiepingensis sp. n.

Taxon classificationAnimaliaAraneaeAgelenidae

http://zoobank.org/B4D3118D-E2CA-4711-BF8F-52BC4DE810B5

Figs 7 , 8 , 11

##### Type material.

**Holotype** ♂: China: Hubei: Enshi Prefecture: Xianfeng County: Zhongtangpu Town: Jieping Village, Shangjieping, Xiangjie, a cave without name, N29.61330°, E109.17803°, 1004 m, 17.I.2014, Y. Li and J. Liu leg. **Paratypes**: 4♀1♂, same data as holotype.

##### Etymology.

The specific name is derived from the type locality; adjective.

##### Diagnosis.

**Figure 7.**
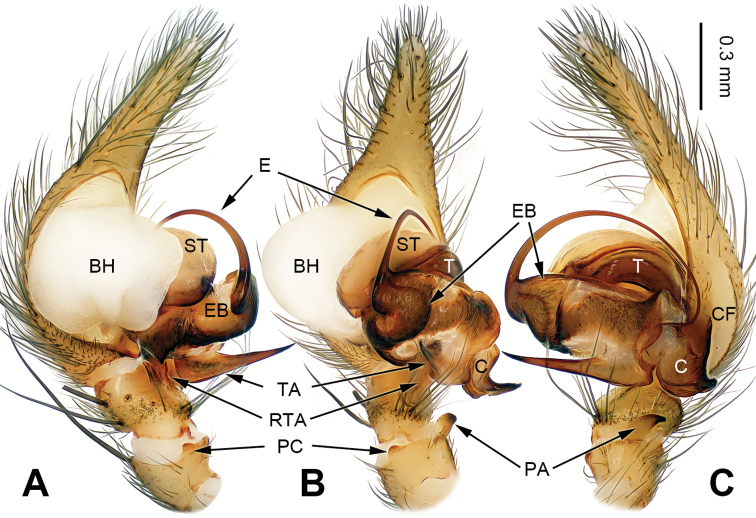
Left male palp of *Papiliocoelotes jiepingensis* sp. n. (expanded), holotype. **A** Prolateral view **B** Ventral view **C** Retrolateral view. Scale bar: equal for **A, B, C**.

**Figure 8.**
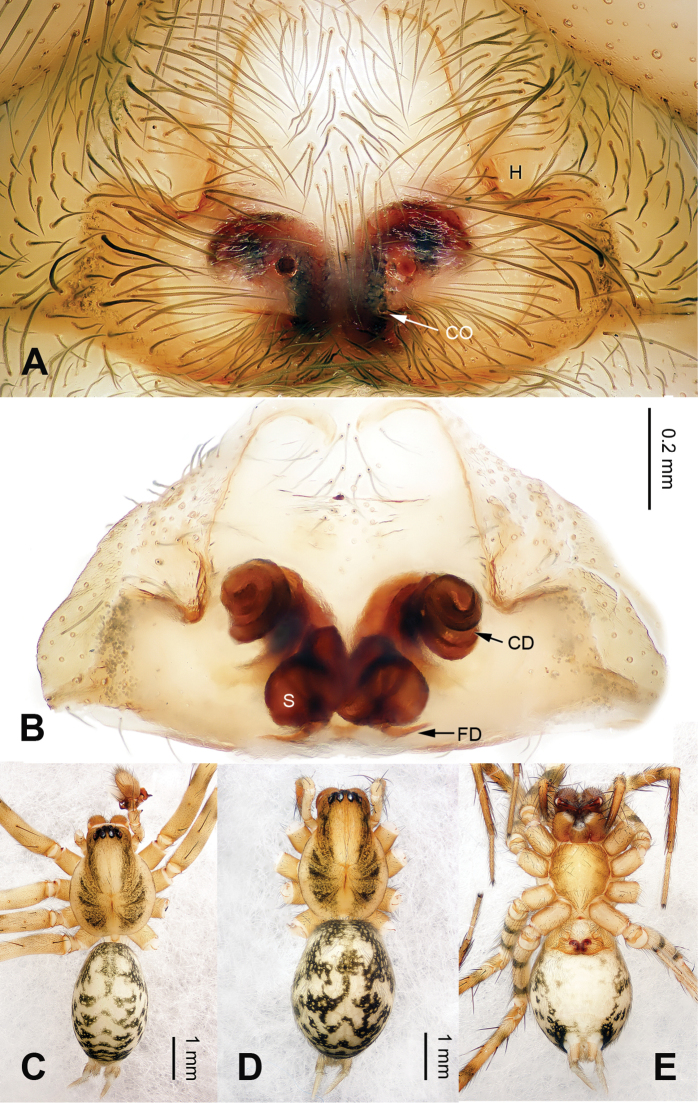
Epigyne and habitus of *Papiliocoelotes jiepingensis* sp. n. **A** Epigyne, ventral **B** Vulva, dorsal **C** Male habitus, dorsal **D** Female habitus, dorsal **E** Female habitus, ventral. Scale bars: equal for **A** and **B**, equal for **D** and **E**.

The male can be distinguished from *Papiliocoelotes yezhouensis* sp. n. by the slender, needle-like tegular apophysis, the short patellar condyle and the flat conductor with 1 pointed retrolateral process (Fig. 7A–C). The female can be distinguished from *Papiliocoelotes yezhouensis* sp. n. by the thin and coiled copulatory ducts, the width of copulatory ducts obviously wider than the spermathecae (Fig. 8A–B).

##### Description.

**Male (holotype)**: Total length 5.45. Carapace 2.55 long, 2.00 wide. Abdomen 2.85 long, 1.80 wide. Eye sizes and interdistances: AME 0.10, ALE 0.13, PME 0.13, PLE 0.14; AME-AME 0.03, AME-ALE 0.02, ALE-PLE 0.03, PME-PME 0.07, PME-PLE 0.08. Leg measurements: I: 9.75 (2.55, 3.20, 2.40, 1.60); II: 8.85 (2.50, 2.85, 2.00, 1.50); III: 8.20 (2.25, 2.45, 2.10, 1.40); IV: 11.40 (3.00, 3.50, 3.15, 1.75). Chelicerae with 2 promarginal teeth. Palp: patellar apophysis short, extending anteriorly, with curved, pointed tip; patellar condyle short; retroventral tibial apophysis small; cymbial furrow short but obvious, about 1/3 length of cymbium; conductor flat with 1 pointed retrolateral process; tegular apophysis slender, needle-like and longer than the length of cymbial furrow (Fig. 7A–C).

**Female (one of paratypes)**: Total length 5.50. Carapace 2.65 long, 1.80 wide. Abdomen 2.90 long, 2.10 wide. Eye sizes and interdistances: AME 0.09, ALE 0.11, PME 0.12, PLE 0.13; AME-AME 0.04, AME-ALE 0.03, ALE-PLE 0.04, PME-PME 0.07, PME-PLE 0.08. Leg measurements: I: 7.90 (2.10, 2.75, 1.80, 1.25); II: 7.20 (2.00, 2.40, 1.65, 1.15); III: 6.90 (1.95, 2.10, 1.85, 1.00); IV: 9.20 (2.45, 3.00, 2.50, 1.25). Chelicerae like in male with 2 promarginal teeth. Epigyne: copulatory openings located posteromedially; epigynal hoods located mediolaterally on epigynal plate, sulci rounded and deep; copulatory ducts thin and coiled with more than 2 loops; the width of copulatory ducts obviously wider than the spermathecae (Fig. 8A–B).

##### Distribution.

Known only from the type locality (Fig. 11).

#### Papiliocoelotes meiyuensis sp. n.

Taxon classificationAnimaliaAraneaeAgelenidae

http://zoobank.org/10118C37-ACDA-4D50-9B4C-14E499048F90

Figs 9 , 10 , 11

##### Type material.

**Holotype** ♂: China: Hubei: Enshi Prefecture: Hefeng County: Zouma Town: Meiyuping, Xini Village, Xianren Cave, N29.73239°, E110.31914°, 853 m, 10.I.2014, Y. Li and J. Liu leg. **Paratypes**: 2♀1♂, same data as holotype.

##### Etymology.

The specific name is derived from the type locality; adjective.

##### Diagnosis.

**Figure 9.**
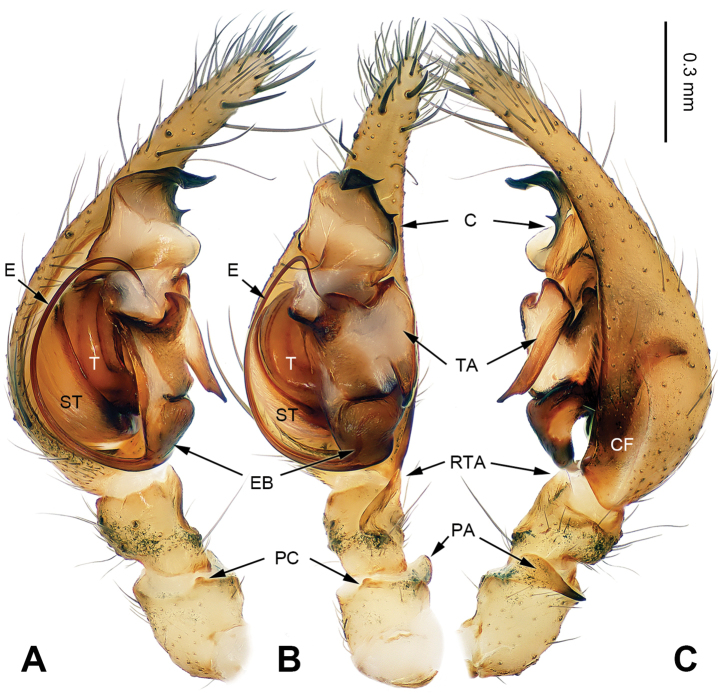
Left male palp of *Papiliocoelotes meiyuensis* sp. n., holotype. **A** Prolateral view **B** Ventral view **C** Retrolateral view. Scale bar: equal for **A**, **B**, **C**.

**Figure 10.**
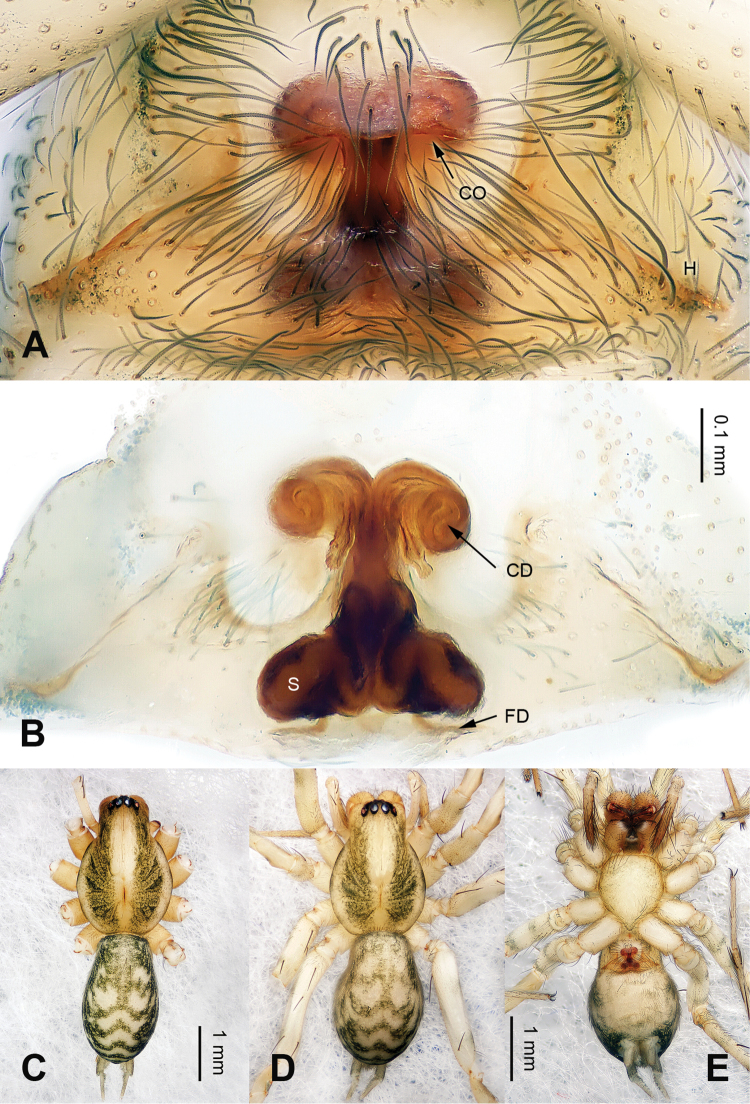
Epigyne and habitus of *Papiliocoelotes meiyuensis* sp. n. **A** Epigyne, ventral **B** Vulva, dorsal **C** Male habitus, dorsal **D** Female habitus, dorsal **E** Female habitus, ventral. Scale bars: equal for **A** and **B**, equal for **D** and **E**.

The male can be distinguished from *Papiliocoelotes yezhouensis* sp. n. by the sharply pointed retroventral tibial apophysis, the shorter patellar condyle, the dorsally extending patellar apophysis, the tegular apophysis with pointed tip and the large conductor with 1 distal process and 1 small spine-like retrolateral process (Fig. 9A–C). The female can be distinguished from *Papiliocoelotes yezhouensis* sp. n. by the distinct copulatory openings, the epigynal hoods located posterolaterally and by the width of the copulatory ducts, which are narrower than the spermathecae (Fig. 10A–B).

##### Description.

**Male (holotype)**: Total length 4.98. Carapace 2.55 long, 1.80 wide. Abdomen 2.40 long, 1.45 wide. Eye sizes and interdistances: AME 0.08, ALE 0.12, PME 0.13, PLE 0.13; AME-AME 0.03, AME-ALE 0.02, ALE-PLE 0.03, PME-PME 0.05, PME-PLE 0.05. Leg measurements: I: 10.20 (2.75, 3.25, 2.35, 1.85); II: 9.00 (2.40, 2.90, 2.10, 1.60); III: 8.65 (2.35, 2.50, 2.35, 1.45); IV: 11.75 (3.00, 3.50, 3.40, 1.85). Chelicerae with 3 promarginal teeth. Palp: patellar apophysis long, slightly curved, with pointed tip, extending dorsally; patellar condyle short; retroventral tibial apophysis sharp pointed, extending beyond the tibia anteriorly; cymbial furrow small, about 1/3 length of cymbium; conductor large, with 1 distal process and 1 small spine-like retrolateral process; tegular apophysis relatively short, with pointed tip and subequal to the length of cymbial furrow (Fig. 9A–C).

**Female (one of paratypes)**: Total length 4.13. Carapace 2.10 long, 1.50 wide. Abdomen 2.10 long, 1.40 wide. Eye sizes and interdistances: AME 0.05, ALE 0.11, PME 0.11, PLE 0.12; AME-AME 0.02, AME-ALE 0.02, ALE-PLE 0.03, PME-PME 0.05, PME-PLE 0.04. Leg measurements: I: 6.60 (1.95, 2.25, 1.50, 0.90); II: 6.05 (1.75, 1.95, 1.30, 1.05); III: 5.90 (1.55, 1.85, 1.55, 0.95); IV: 7.65 (2.25, 2.45, 2.10, 0.85). Chelicerae with 2 promarginal teeth. Epigyne: copulatory openings located centrally; epigynal hoods located posterolaterally, sulci round; copulatory ducts small and spiraled; the width of the copulatory ducts narrower than the spermathecae (Fig. 10A–B).

##### Distribution.

**Figure 11.**
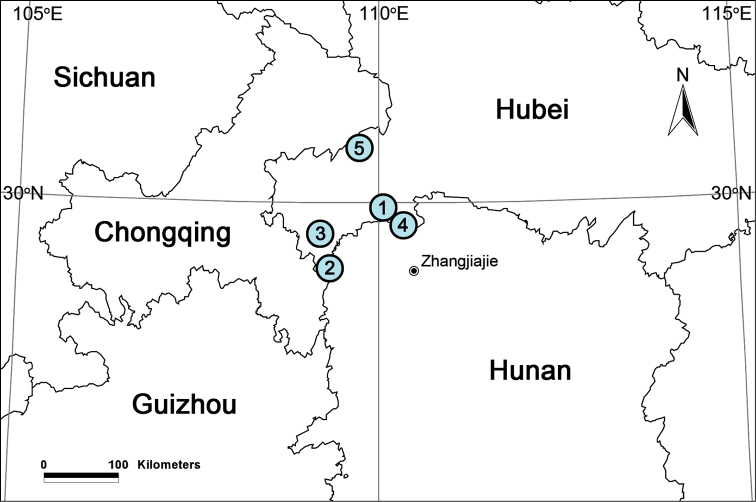
Localities of new *Papiliocoelotes* species from China. **1** *Papiliocoelotes guanyinensis* sp. n. **2** *Papiliocoelotes guitangensis* sp. n. **3** *Papiliocoelotes jiepingensis* sp. n. **4** *Papiliocoelotes meiyuensis* sp. n. **5** *Papiliocoelotes yezhouensis* sp. n.

Known only from the type locality (Fig. 11).

## Supplementary Material

XML Treatment for Papiliocoelotes

XML Treatment for Papiliocoelotes yezhouensis

XML Treatment for Papiliocoelotes guanyinensis

XML Treatment for Papiliocoelotes guitangensis

XML Treatment for Papiliocoelotes jiepingensis

XML Treatment for Papiliocoelotes meiyuensis
